# The involvement of underrepresented countries in the European rare diseases research alliance: Morocco’s assets

**DOI:** 10.1186/s13023-025-03965-0

**Published:** 2025-11-24

**Authors:** Naima Fdil

**Affiliations:** Cadi Ayyad University, UCA, Faculty of Medicine and Pharmacy, Metabolic platform, Biochemistry Laboratory, Research center: Childhood, Health and Sustainable Development. , B.P. 7010, Marrakech, Morocco

**Keywords:** Rare diseases, ERDERA, Underrepresented countries, Morocco

## Abstract

Rare diseases (RDs) pose unique challenges to global healthcare. The European Union has led numerous initiatives, including EURORDIS, European Reference Networks (ERNs), and recently, the European Rare Diseases Research Alliance (ERDERA), to advance research, diagnosis, and treatment in this field. A crucial aspect of ERDERA is the strategic involvement of underrepresented countries (UCs), acknowledging their unique contributions to RD research and capacity building.

This paper takes Morocco as a case study to explore the scientific and translational benefits that UCs bring to ERDERA. With high rates of consanguinity, large groups of untreated patients, and the emergence of specific or novel mutations, these countries are positioned as key allies in discovering pathogenic variants, creating therapeutic targets, and enhancing precision diagnostics. We argue that integrating UCs into RD research frameworks not only strengthens the scientific ecosystem but also promotes equity, capacity development, and mutual benefit across regions.

Our findings support a shift toward inclusive and internationally coordinated strategies, emphasizing that the success of RD initiatives such as ERDERA depends on leveraging global diversity and fostering North–South collaborations.

## Background

Rare diseases (RDs) are defined in Europe as those affecting no more than 5 people per 10,000. Though each is individually uncommon, they collectively affect an estimated 30 million people in the EU. Globally, there are between 5,000 and 7,000 identified rare diseases, many of which are chronic, debilitating, and often genetic in origin. Most lack effective treatments or even accurate diagnoses, largely due to insufficient research and limited knowledge of their underlying mechanisms.

This challenge is not unique to Europe. Countries like the United States and Japan have also recognized rare diseases as public health priorities. The U.S. introduced the Orphan Drug Act in 1983 to incentivize research and development, while Japan has implemented similar national policies. More recently, low- and middle-income countries are beginning to recognize the burden of rare diseases, though many still lack the infrastructure to address them adequately [1]. 

The 2019 UN resolution officially acknowledged people living with rare diseases as among the most vulnerable populations excluded from universal health coverage, reinforcing the global need for collaborative action and investment in rare disease research and care.

In consideration of this context, and with the support of the European Union, many actors in Europe have contributed to the establishment of important initiatives to address the challenges posed by rare diseases. Some of the milestones included:

- The creation of the patient’s organizations alliance (EURORDIS), a non-profit alliance of patient organizations and individuals dedicated to improving the lives of people affected by rare diseases across Europe. Founded in 1997 by four patient groups, EURORDIS serves as a unifying voice for patients, promoting research, better care, and policy change. It is governed by a board elected from its member organizations and focuses on empowering patients, supporting scientific collaboration, advocating at various political levels, and fostering online communities like RareConnect. With nearly 600 member organizations, EURORDIS plays a critical role in advancing diagnosis, treatment, and social support for the rare disease community [2].
- The creation of the International Rare Disease Research Consortium (IRDiRC) in 2011 by the European Commission and the U.S. National Institutes of Health to coordinate and amplify rare disease research worldwide. Bringing together funders, patient groups, scientists, industry leaders, and regulators, IRDiRC aims to overcome the challenges posed by the limited commercial interest in rare diseases. Initially, IRDiRC focused on two key objectives: enabling the development of 200 new therapies and improving diagnostic capabilities for rarest diseases by 2020; goals it helped surpass ahead of schedule. Building on this momentum, the consortium adopted a new vision for 2017–2027: ensuring timely diagnosis and treatment access within one year of medical consultation, approving 1,000 new therapies (especially for untreated conditions), and developing methods to evaluate the real-world impact of these advancements on patients’ lives [3].
- The implementation of the European Reference Networks (ERNs) in 2017, are collaborative networks connecting specialized healthcare providers across EU Member States to improve access to expertise and treatment for complex or rare conditions. Each ERN must include at least ten healthcare centers from eight different countries, aiming to overcome the challenges posed by low patient numbers, limited expert knowledge, and scarce resources in individual countries. Established under specific EU regulations, ERNs ensure that no country has to address highly specialized medical needs alone. Unlike local or border-region healthcare agreements, ERNs operate EU-wide, enabling collaboration beyond national or regional boundaries [4].
- The European Platform on Rare Disease Registration (EU RD Platform) is a European Commission-led initiative designed to reduce the fragmentation of rare disease (RD) patient data across Europe, which has traditionally been dispersed among hundreds of disconnected registries. Central to this effort is the European Rare Disease Registry Infrastructure (ERDRI), launched in 2018, the RD Platform fosters interoperability and harmonization across RD data sources by promoting common data elements and detailed metadata standards. This infrastructure enables both the integration of existing registries and the development of new ones, thereby supporting large-scale studies essential for progress in areas such as epidemiology, clinical care, translational research, and orphan drug development [5].

Efforts to improve interoperability and collaboration among rare disease registries have gained momentum through both technological innovations and national strategies. The findings point to an urgent need for a nationally and internationally coordinated, systematic approach to unify efforts and ensure consistent, high-quality data across registries. Such activities illustrate how interoperability frameworks and strategic planning can enhance registry collaboration, data quality, and ultimately, outcomes for individuals living with rare diseases [6, 7].

This awakening within the European Union laid the groundwork for the launch of The European Joint Program on Rare Diseases (EJP RD) in 2019 That plays a pivotal role in advancing rare disease research by bringing together more than 130 organizations from 35 different countries. Its primary goal is to bridge the gap between fundamental research and real-world clinical solutions through international collaboration and strong involvement of patient communities. Through its efforts, the programme has supported diverse research initiatives, launched a digital platform for rare diseases, and made notable contributions in training, project support, and the development of innovative research methodologies [8]. 

All of these initiatives aim to establish effective research on rare diseases (RDs) and promote progress and innovation in diagnosis and patient care.

In September 2024, the European Rare Diseases Research Alliance (ERDERA) was launched [9]. The ERDERA is a collaborative initiative that aimed at strengthening and integrating research efforts across Europe to address the unmet needs of people living with rare diseases. ERDERA fosters partnerships among national and international research institutions, healthcare providers, patient organizations, and industry stakeholders to advance the diagnosis, treatment, and care of rare disease patients. It plays a key role in promoting data sharing, harmonizing research agendas, and supporting the use of emerging technologies like genomics and artificial intelligence. The alliance works in close synergy with the European Joint Programme on Rare Diseases (EJP RD), and contributes to the goals of the International Rare Diseases Research Consortium (IRDiRC).

The ERDERA has the capacity to involve underrepresented countries (UCs) in its initiatives, fostering a more equitable and inclusive European research ecosystem. By engaging countries with limited rare disease infrastructure or visibility in research, ERDERA helps strengthen local capacities through training, resource sharing, and participation in transnational projects. It capitalizes on the unique strengths these countries may offer, such as genetic diversity, population-based data, and emerging clinical networks, while supporting the development of national rare disease strategies and registries [10]. 

### Under-represented countries (UCs) in the context of rare diseases

UCs are generally those with limited visibility, participation, or influence in international health policy, research, and funding related to rare conditions. These countries typically fall into one or more of the following categories: (a) Low- and Middle-Income Countries, (b) Countries with weak Health and Research Infrastructure, (c) Politically or Economically Isolated Nations and (d) Neglected in International Data Sets. These countries often face critical challenges, including a lack of newborn screening, inadequate research funding for rare diseases, limited access to orphan drugs, and an underdeveloped patient advocacy infrastructure.

Thanks to the implementation of National Contact Points (NCPs) in several underrepresented countries as part of the Horizon Europe framework programme, some of these nations have successfully become active partners in the European Rare Diseases Research Alliance (ERDERA). These partnerships represent a significant step forward in addressing disparities in rare disease research and fostering international collaboration. Thus, countries such as Bulgaria, Cyprus, Czechia, Estonia, Georgia, Greece, Hungary, Iceland, Latvia, Lithuania, Turkey, Poland, Portugal, Romania, Serbia, Slovakia, Slovenia, and Morocco, have joined ERDERA.

To highlight the significant role of UC involvement in the ERDERA, we take Morocco as an example. Morocco is similar to many other countries that lack representation, encounters various financial, educational, infrastructural, and operational difficulties that greatly impede the progress and execution of effective strategies for rare diseases.

However, since 2016, there has been a remarkable shift in awareness and engagement surrounding these conditions in the country. A pivotal moment in this journey was the creation of the Metabolic Platform at the Faculty of Medicine and Pharmacy, Cadi Ayyad University. This platform, which also hosts the Moroccan Association for Inherited Metabolic Diseases, has become both a physical center and a scientific driving force. Over the past eight years, it has played a vital role in diagnosing and managing more than 200 confirmed cases of inherited metabolic disorders, such as mucopolysaccharidoses, Wilson disease, and metachromatic leukodystrophy [11, 12], using a combination of clinical evaluations, biochemical tests, and next-generation sequencing.

In this paper, we report Morocco’s specific holdings on the basis of our experience and daily practices during the last ten years with Moroccan patients suffering from rare diseases. The Morocco assets related to rare diseases, to be considered and capitalized, are numerous and diverse.

### Assets of underrepresented countries: a focus on Morocco

#### Elevated consanguineous marriage rate

The elevated consanguineous marriage rate in Morocco is a double-edged sword. While it increases the prevalence of rare diseases, it also presents unique opportunities for advancing their diagnosis and treatment. By leveraging these populations’ genetic insights and implementing targeted interventions, healthcare systems can turn a genetic challenge into a platform for medical innovation and improved patient care. Several previous studies have highlighted the significant impact of consanguineous marriages on the prevalence and spectrum of genetic diseases in Morocco. They have also highlighted the role of consanguinity in the expression of rare diseases in the population [13, 14]. This practice means that Morocco has consistent cohorts of patients with or suspected of having RD, which may help alleviate the limitations imposed by the rarity of affected patients, leading to limited knowledge and expertise [15] and hampering the commitment of treatment developers [16].

#### The high number of untreated patients suffering from treatable RD

Diagnostic delays and the limited availability of specific treatments at the national level have led to the existence of large cohorts of patients diagnosed with treatable rare diseases, without being able to benefit from it for several reasons. While this situation presents considerable challenges, it also opens the door to some potential opportunities and benefits for programs such as ERDERA:

- Enhancing the understanding of disease progression is particularly valuable in untreated populations, as they provide a unique opportunity to observe the natural history of rare diseases [17]. Studying such populations can yield critical insights into disease onset, symptom evolution, and long-term outcomes. This approach helps identify phenotypic variability and natural disease trajectories, which can in turn refine diagnostic criteria and inform the development of more effective, targeted, and personalized treatment strategies [18].
- Supporting the identification of therapeutic targets is a key advantage of studying untreated rare disease populations. Without the influence of prior treatments, researchers can observe the disease in its unaltered, “pure” form, allowing for the identification of underlying molecular or cellular mechanisms that may serve as potential targets for therapy [19]. Additionally, these untreated cohorts offer valuable opportunities to uncover biomarkers that facilitate early diagnosis or help monitor disease progression, ultimately contributing to the development of more precise and effective treatments [20, 21].
- Facilitating clinical trials: Large untreated rare disease populations play a crucial role in accelerating therapeutic development. First, they offer a valuable pool of participants for clinical trials, enabling the robust evaluation of new, potentially more effective and accessible therapies [22]. Second, they can serve as natural history-based control groups, particularly in trials where using placebos may be unethical or impractical. These untreated cohorts help researchers assess treatment efficacy by providing meaningful comparisons to the natural progression of the disease [23].
- Offering insights that might not be evident in treated populations in developed countries especially the likely interactions with different ecosystems, such as COVID-19 infection [16, 24].

#### Recurrence of a specific mutation in a given rare disease

Both the broader population and isolated Moroccan communities, shaped by geographic isolation, ancestral customs, and unique ecosystems, offer invaluable opportunities for advancing rare disease research. These communities support: a) rapid and conclusive genotype‒phenotype correlation studies, which accelerate diagnosis and contribute to the development of precision medicine [25], deeper understanding of genetic mechanisms, including the role of founder effects, consanguinity, and environmental influences in disease expression [26], and insight into epigenetic contributions to rare diseases, offering new perspectives for both innovative treatments and preventive strategies.

#### The discovery of new mutations causing a rare disease

The identification of new mutations and/or the confirmation of the pathogenicity of mutations previously described as “pathogen-like” [27, 28] offer several critical contributions to the fields of rare disease research, diagnostics, and treatment in several ways:

- Advancing genetic diagnostics by (a) identifying new causative mutations, reducing diagnostic errors and the time to diagnosis for affected individuals [29]; (b) expanding genetic panels [30]; and (c) helping in distinguishing between benign genetic variations and disease-causing mutations, refining diagnostic criteria [31].
- Enhancing the understanding of disease mechanisms [32, 33] by: (a) providing insight into rare disease pathophysiology, (b) identifying key genes contributing to a deeper understanding of the roles that specific genes play in the onset and progression of rare diseases, laying the groundwork for targeted research, and (c) uncovering phenotypic variability.
- Supporting drug development and therapeutics [34, 35] by (a) enabling the identification of more molecular targets for therapeutic development and (b) repurposing existing drugs: Confirmation of mutation pathogenicity can lead to the identification of existing drugs that may be effective for newly characterized genetic subtypes of a disease.

#### The absence of a national plan for rare diseases

This has several critical consequences, including (a) the absence of national reference laboratories causing late diagnosis and misdiagnosis and a lack of genetic counseling, (b) the need for a governance framework and coordination structure to increase awareness of the role of consanguineous marriages in the incidence of rare diseases, and (c) the lack of theoretical and practical training among health professionals (medical and paramedical). Given these conditions and circumstances, the number of patients with rare diseases is high, the number of families with more than one affected patient by the same rare disease is high, and the number of patients suffering from more than one rare disease is also high [12]. Involving such cohorts in research programs and/or clinical trials could be beneficial both, for the patient and for the rare disease ecosystem.

Stakeholders of previous programs dedicated to supporting research and innovation in RDs have concluded, among other things, that for most understudied rare diseases, the size of the patient population at the national or even continental level is insufficient to generate the critical mass of knowledge on the disease and attract the interest of drug developers, that progress towards the international interoperability of clinical research networks is crucial to better understand rare diseases [9, 36]. The involvement of UCs in ERDERA activities is, therefore, an action that capitalizes on the recommendations issued by previous programs and will ensure the achievement of ERDERA’s objectives and, at the same time, contribute to improving the overall context of diagnosis and management of patients with rare diseases.

## Conclusion

Advancements in rare disease (RD) research and care rely heavily on collaborative efforts that go beyond borders, disciplines, and resource differences. International partnerships and the use of diverse data sources, particularly registries, are crucial in overcoming challenges like low prevalence, genetic variations, and delays in diagnosis. By strengthening global networks, we can speed up genotype-phenotype correlation studies, improve diagnostics, and develop personalized treatments. The integration of underrepresented countries (UCs) into the European Rare Diseases Research Alliance (ERDERA) plays a key role in enhancing our understanding of genetic, environmental, and epigenetic diversity. This integration not only benefits research, diagnostics, and treatments for RD patients but also creates opportunities for inclusive collaboration and knowledge sharing. It strengthens ERDERA’s position as a strategic and fair platform for international cooperation in the field of rare diseases.

## Data Availability

Not applicable.

## Abbreviations

RDs: Rare Diseases
EURORDIS: Europe rare diseases patient’s organizations alliance
IRDiRC: International Rare Diseases Research Consortium
ERNs: European Reference Networks
EJP RD: The European Joint Program on Rare Diseases
ERDERA: The European Rare Diseases Research Alliance
UCs: Under represented Countries
